# Supplementary material for: A CDK-regulated chromatin segregase promoting chromosome replication
Source: Nat Commun. 2021 Sep 1;12:5224. doi: 10.1038/s41467-021-25424-7 (PMC8410769; doi:10.1038/s41467-021-25424-7)
Supplement: Supplementary file 1 — Supplementary Information [file 41467_2021_25424_MOESM1_ESM.pdf]

Supplementary Information

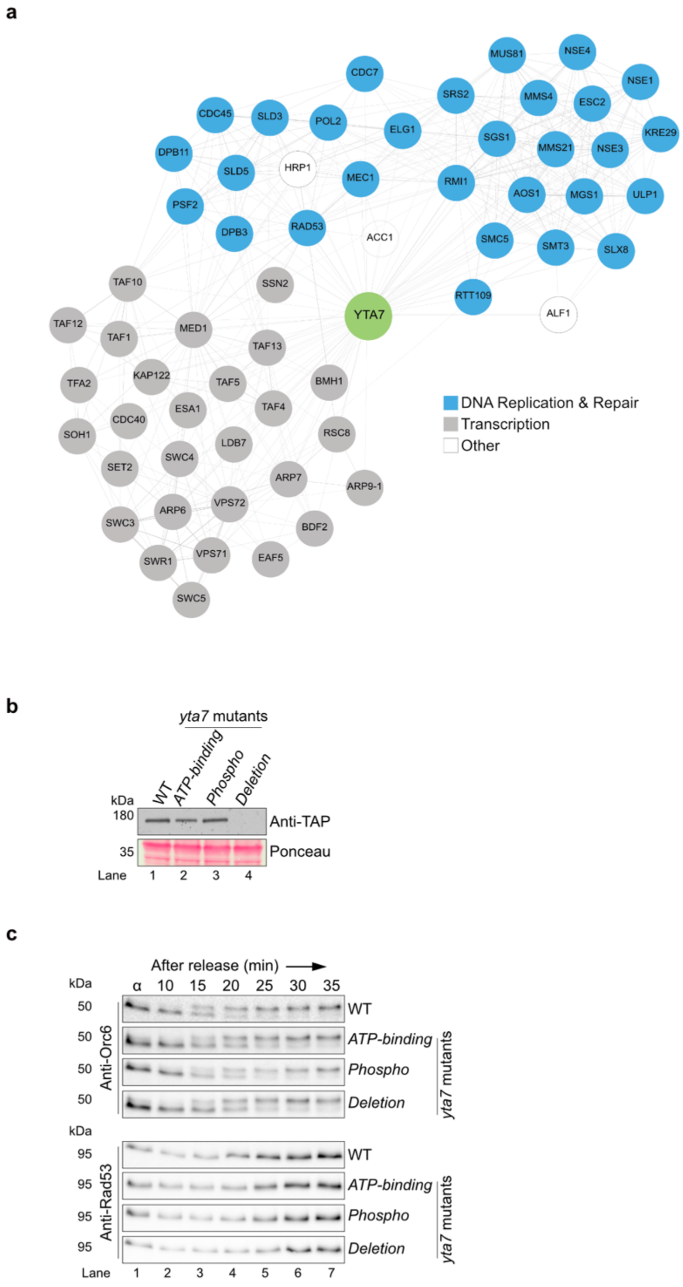

Supplementary Fig. 1. Yta7 is involved in chromosome replication *in vivo*

a) Genes showing similar genetic interaction profiles compared to *YTA7*.

b) Expression levels of cells bearing Yta7 wild-type (WT), *yta7* AAA^{+}-ATPase- (*ATP-binding*), *yta7* CDK-phospho- (*Phospho*) mutations. *yta7Δ* (*Deletion*) cells were included as a control. Cells were grown to early log-phase, proteins were precipitated using trichloroacetic acid and analysed by immunoblotting.

c) Strains as in **b** were synchronised in G1 phase with alpha-factor, released into fresh medium and Rad53 activation and Orc6 phosphorylation were analysed by immunoblotting at indicated time points. Shown are representative experiments, which have been biologically replicated three times.

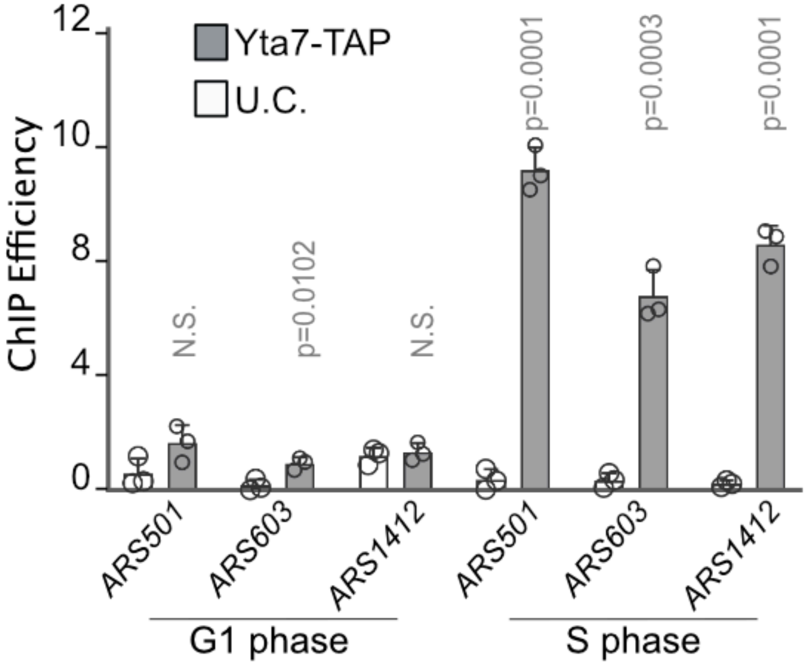

Supplementary Fig. 2. Yta7 localises to late replication origins during early S phase.

Cells bearing Yta7-FLAG were synchronised in G1 phase using alpha factor, released and harvested during early S phase. Chromatin-Immunoprecipitation (ChIP) experiments were performed as described in Methods. U.C. means untagged control. Mean values and standard deviations (S.D.) were obtained from three biological replicates. P-values were obtained by using two-tailed unpaired *t*-test calculations. N.S. means statistically non-significant.

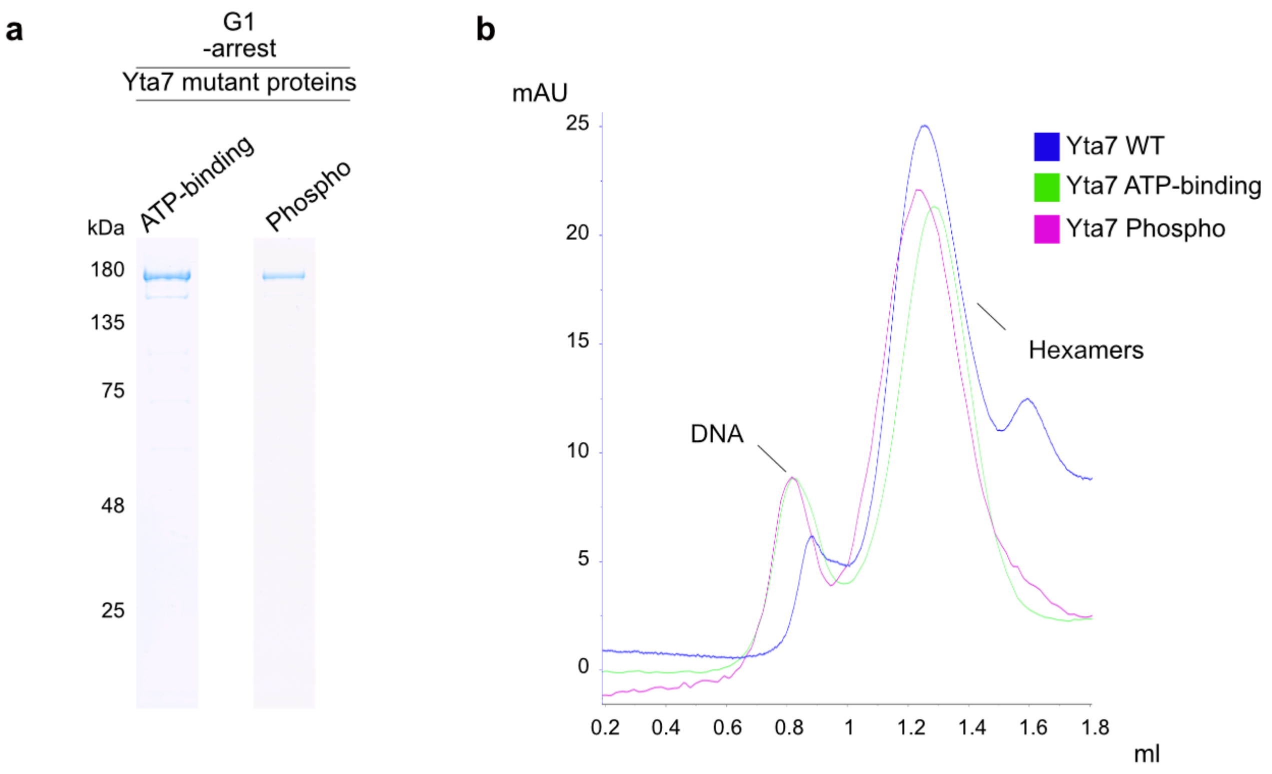

Supplementary Fig. 3. Yta7 is a AAA^{+}-ATPase, related to molecular segregases

a) Purified Yta7 AAA+-ATPase-(ATP-binding) and CDK phospho-mutant (Phospho) from G1 phase cells analysed by SDS-PAGE with Coomassie-staining.

b) Size exclusion chromatography profiles of Yta7 WT and Yta7 ATP-binding *and* Phospho mutants. mAU (milli absorbance unit) means protein absorbance at 280 nM.

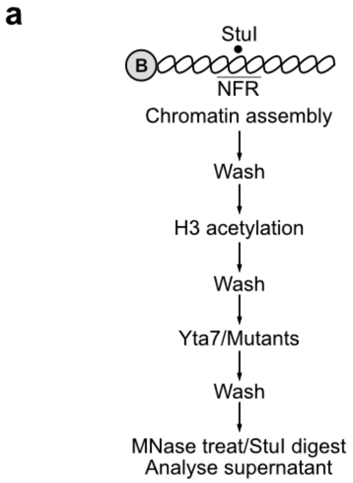

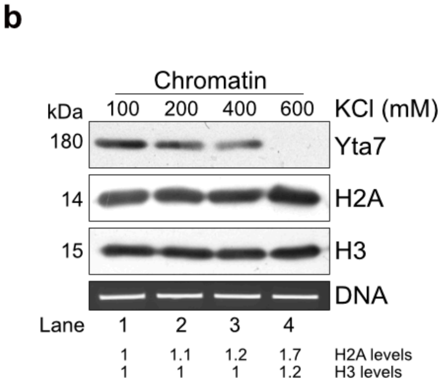

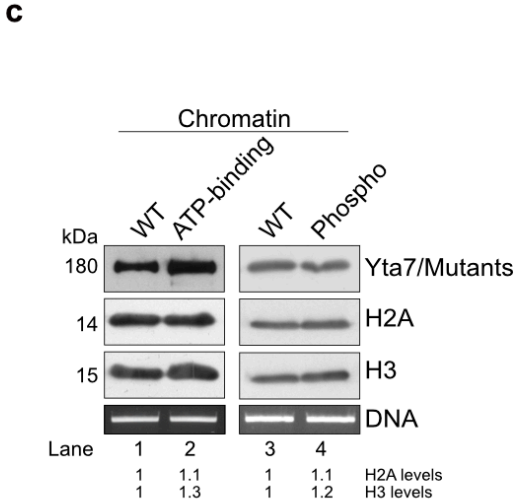

Supplementary Fig. 4. Yta7 recruitment to chromatin

a) Scheme of recruitment assay of Yta7.

b) Yta7 recruitment was monitored after washes with indicated KCl concentrations. H2A, H3 and Yta7 were analysed by immunoblotting. StuI digested DNA by agarose gel electrophoresis as in **Fig. 3a** and **b**.

c) Recruitment of Yta7 ATP-binding- and Phospho-mutants was compared to Yta7 WT. H2A, H3 and Yta7 levels were analysed by immunoblotting. StuI digested DNA was visualised by agarose gel electrophoresis as in **Fig. 3a** and **b**. Shown are representative experiments, which have been biologically replicated three times.

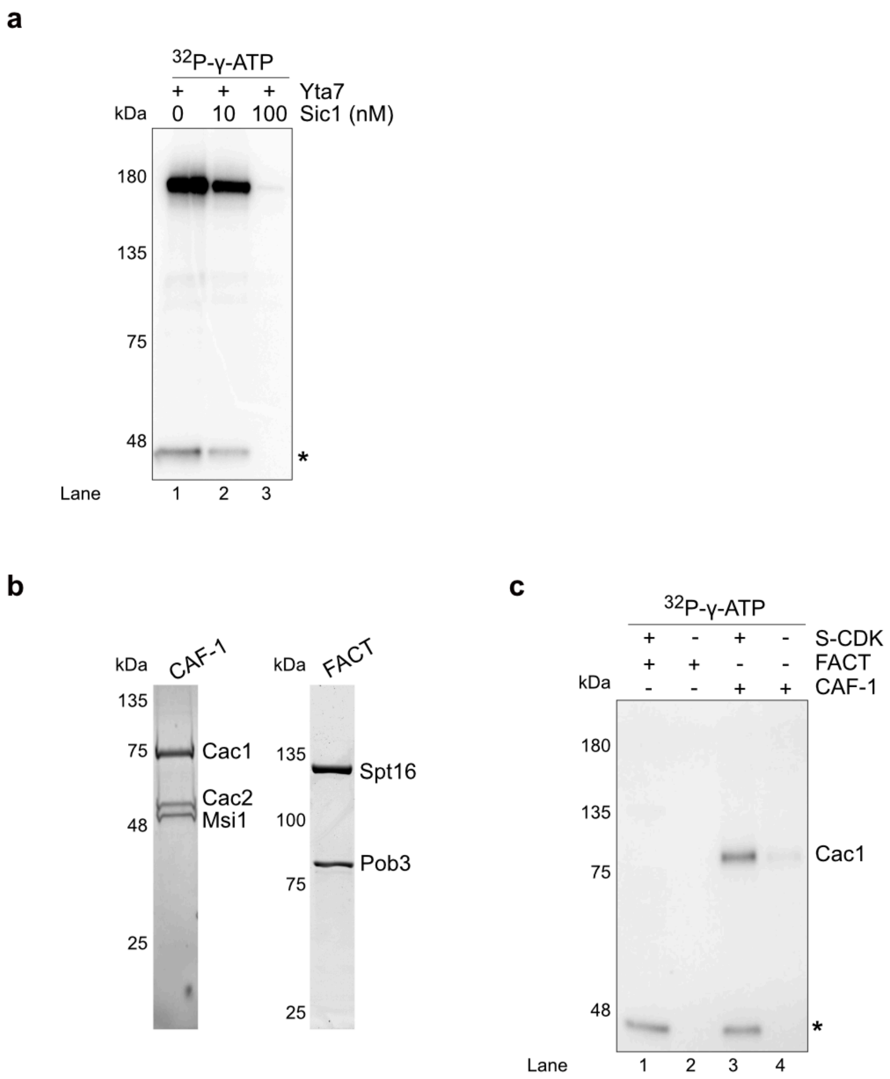

Supplementary Fig. 5. Yta7 is a target of S-CDK

a) *In vitro* kinase assay as in **Fig. 4b** with increasing amounts of Sic1.

b) Purified CAF-1 and FACT complexes analysed by SDS-PAGE with Coomassie-staining.

c) *In vitro* kinase assay as in **Fig. 4b** with purified CAF-1 and FACT as substrates. Asterisk shows auto-phosphorylation of S-CDK. Shown are representative experiments, which have been biologically replicated three times.

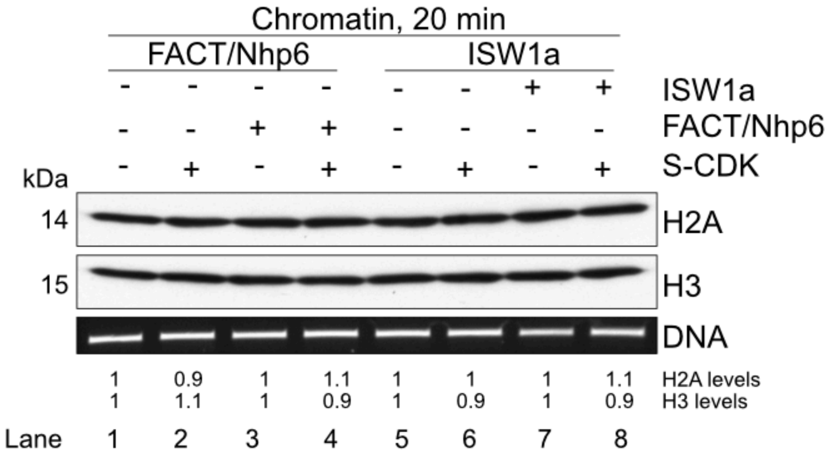

Supplementary Fig. 6. FACT/Nhp6 or ISW1a cannot segregate chromatin.

Chromatin disassembly assay was performed and analysed as in **Fig. 5b** in the presence and absence of FACT/Nhp6, ISW1a and S-CDK. Shown are representative experiments, which have been biologically replicated three times.

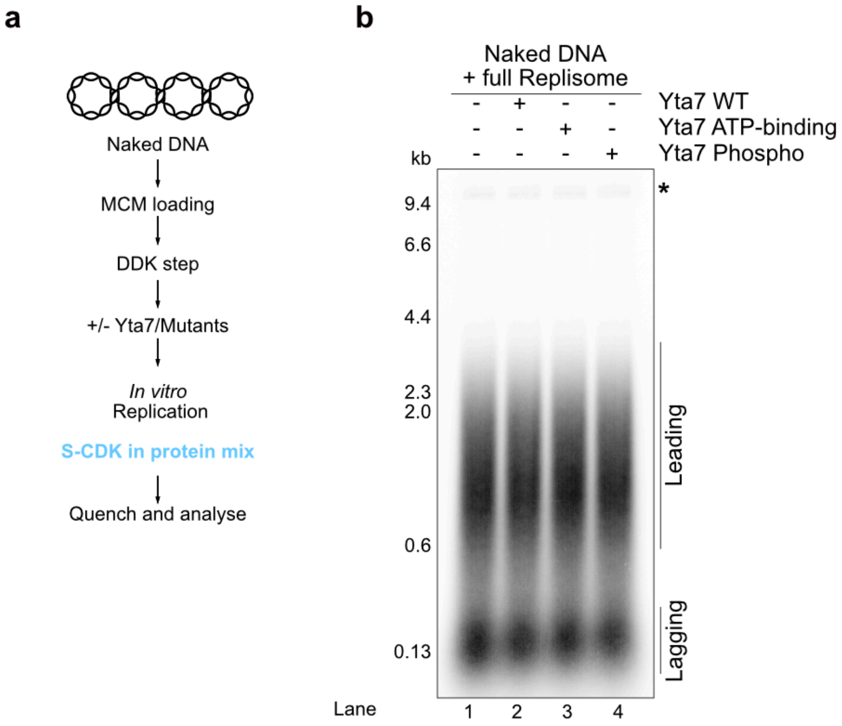

Supplementary Fig. 7. Yta7 does not stimulate replication on naked DNA templates.

a) Scheme of the *in vitro* replication assay on naked DNA.

b) Soluble replication reaction on ARS1-containing 10.6 kb plasmid DNA. MCM loading, DDK treatment and replication reaction as in **Fig. 5e**. Yta7 and mutant proteins were added at the beginning of the reaction. Reactions were stopped after 5 minutes and freshly replicated DNA was visualised by the incorporation of [α-^{32}P] deoxycytidine triphosphate (dCTP) into nascent DNA and products were separated trough 0.8 % alkaline agarose gels and visualised by phosphoimaging. Leading and lagging strands are visible because the assay does not include factors required for Okazaki fragment maturation. Asterisk indicates end labelling of nicked plasmid DNA. Shown are representative experiments, which have been biologically replicated three times.

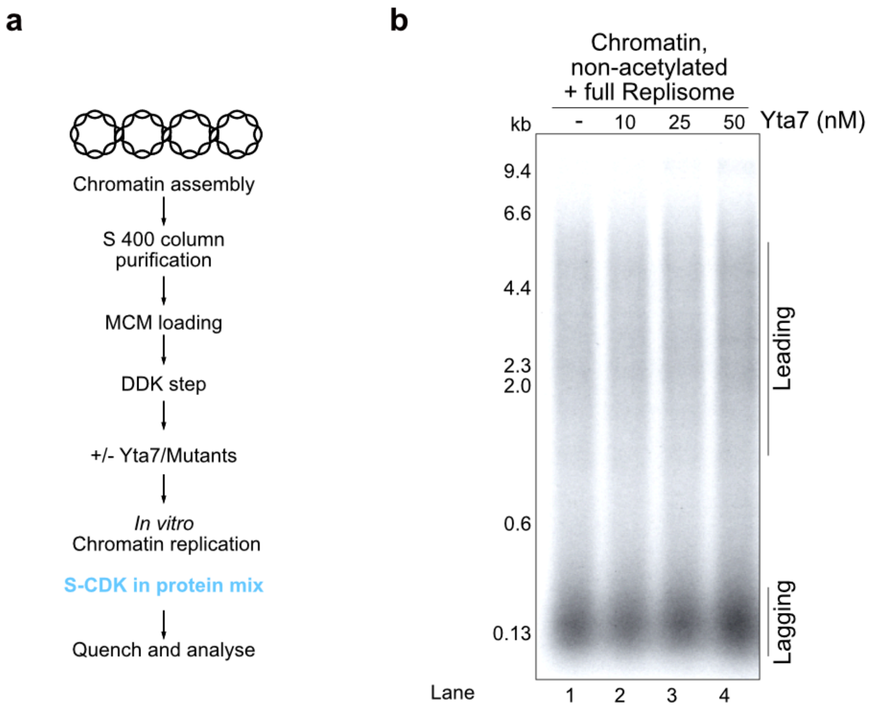

Supplementary Fig. 8. Yta7 does not stimulate replication on chromatin templates without histone H3 acetylation.

a) Scheme of the *in vitro* replication assay on non-acetylated chromatin templates.

b) Soluble replication reaction on ARS1-containing 10.6 kb plasmid DNA. Chromatinisation as in **Fig. 5b** but without H3 acetylation. MCM loading, DDK treatment and replication reaction as in **Fig. 5e**. Shown are representative experiments, which have been biologically replicated three times.

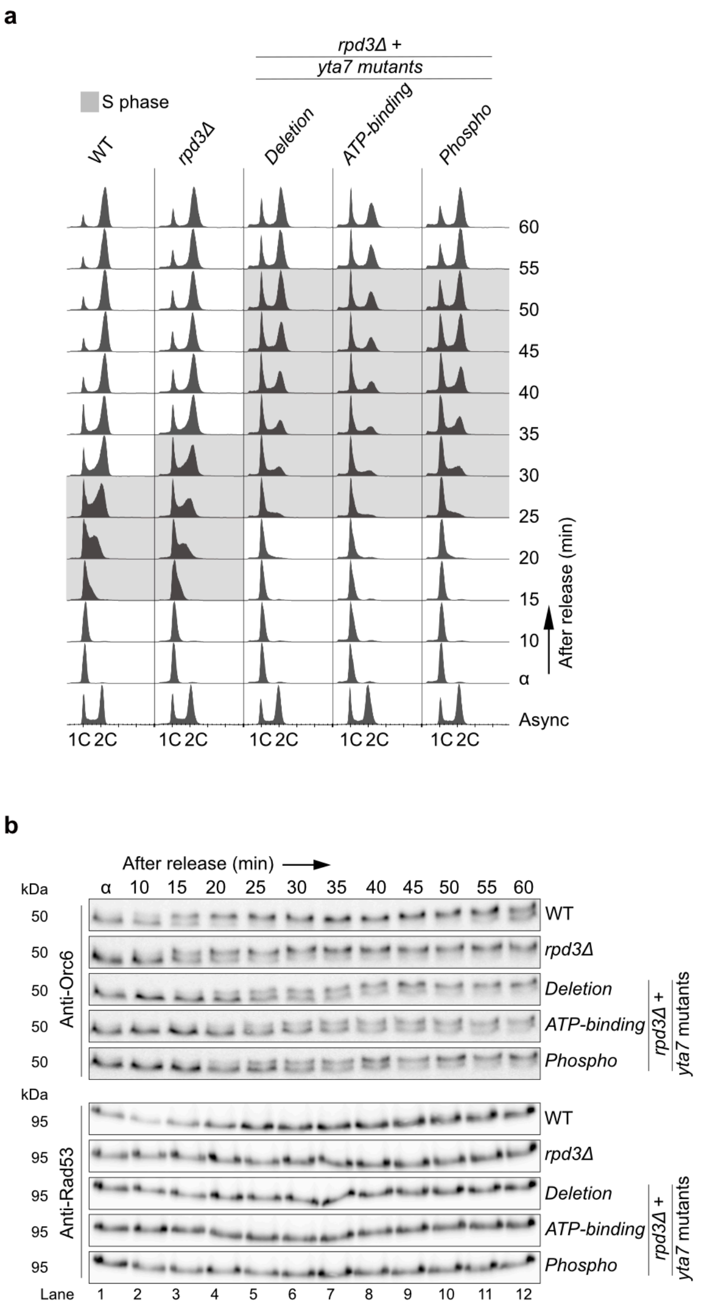

Supplementary Fig. 9. Yta7 is important for S phase entry and progression when histones are hyperacetylated

a) *YTA7* wild-type (WT), cells lacking the major histone deacetylase *RPD3 (rpd3Δ*) and double mutants where *RPD3* was deleted in *yta7 ATP-binding-*, *Phospho-* and *Deletion*-mutants were

synchronised in G1 phase with alpha-factor, released into fresh medium and DNA replication was monitored using FACS. DNA contents 1C and 2C mean non-replicated or replicated DNA, respectively.

b) Protein samples from **a** were taken at indicated time points and checkpoint activation (Rad53 hyper-phosphorylation) as well as Orc6 phosphorylation were analysed by immunoblotting. Shown are representative experiments, which have been biologically replicated three times.

Primers for ChIP assays

Name	Sequence
ARS1-F	5´-CTAAGAAATAGGTTATTACTGAGTAG
ARS1-R	5 -AATCAAAAAGCCAAATGATTTAG
ARS305-F	5´-GTCCAGTTTCATGTACTGTC
ARS305-R	5´-GAGTATTTGATCCTTTTTTTTATTGTG
ARS1426-F	5´-CAAGAATCTGGTTTTACTCG
ARS1426-R	5´- CCGCTATTTTTTTAATTTTAGTTAAG
ARS501-F	5´-CTTTGTGTTACTAATATTATTAATATCTTG
ARS501-R	5´-TACGCATATGCACCATAG
ARS603-F	5´-CATAGACACATTCATAGGTTAAATAG
ARS603-R	5´-GAGGCTAAATTAGAATTTTTGAAG
ARS1412-F	5´-GATTCGTAAAAATTTTTGCAGAC

ARS1412-R	5´- GTAAATATATATACATAAAAGAATGTCGTC

Strains

Yeast strains were generated using standard genetic techniques. SD means codon-optimised.

Strain	Genotype	Source
yCFK-85	*MAT*a, *ura3Δ0, leu2Δ0, his3Δ1, met15Δ0, YTA7-TAP::HIS3*	Andrews lab
yCFK-41	*MAT*a, *ura3Δ0, leuΔ0, his3Δ1, met15Δ 0, yta7-K460A-TAP::HIS3*	Andrews lab
yCFK-52	*MAT*a, *ura3Δ0, leu2Δ0, his3Δ 1, met15Δ0, yta7-7A-TAP::HIS3*	Andrews lab
yCFK-63	*MAT*a, *ura3Δ0, leu2Δ0, his3Δ1, met15Δ 0, yta7::URA3*	This study
yCFK-1	*MAT*a, *ade2-1*, *ura3-1*, *his3-3-11*, *15 trp1-1, leu2-3, 112 can1-100, bar1::Hyg, pep4::KanMX, trp1::TRP1pRS304/SD-Yta7-TEV-3xFLAG*	This study
yCFK-73	*MAT*a, *ade2-1*, *ura3-1*, *his3-3-11*, *15 trp1-1, leu2-3, 112 can1-100, bar1::Hyg, pep4::KanMX, trp1::TRP1pRS304/SD-yta7-ATPase-TEV-3xFLAG*	This study
yCFK-99	*MAT*a, *ade2-1*, *ura3-1*, *his3-3-11*, *15 trp1-1, leu2-3, 112 can1-100, bar1::Hyg, pep4::KanMX, trp1::TRP1pRS304/SD-yta7-Phospho-TEV-3xFLAG*	This study

WFYTA7	*MAT*a*-inc trp1-1 ura3-1 ade2-1 his3-11,15 can1-100 leu2Δ::SFA1 met17Δ::GAL-FLPH305L::HPHMX6 yta7Δ::NATNT2*	This study
WFYK41	*MATα trp1-1 ura3-1 ade2-1 his3-11,15 can1-100 leu2Δ::SFA1 met17Δ::GAL-FLPH305L::HPHMX6 yta7-K460A-TAP::HIS3*	This study
WFYK52	*MAT*a *trp1-1 ura3-1 ade2-1 his3-11,15 can1-100 leu2Δ::SFA1 met17Δ::GAL-FLPH305L::HPHMX6 yta7-13A-TAP::HIS3*	This study
yCFK-54	*MAT*a, *ura3Δ0, leu2Δ0, his3Δ1, met15Δ 0, rpd3::KanMX*	This study
yCFK-57	*MAT*a, *ura3Δ0, leu2Δ0, his3Δ1, met15Δ0, rpd3::KanMX, yta7-K460A-TAP::HIS3*	This study
yCFK-100	*MAT*a, *ura3Δ0, leu2Δ0, his3Δ1, met15Δ0, rpd3::KanMX, yta7-7A-TAP::HIS3*	This study
yCFK-102	*MAT*a, *ura3Δ0, leu2Δ0, his3Δ1, met15Δ0, rpd3::KanMX, yta7::URA3*	This study
