# Supplementary material for: A CDK-regulated chromatin segregase promoting chromosome replication
Source: Nat Commun. 2021 Sep 1;12:5224. doi: 10.1038/s41467-021-25424-7 (PMC8410769; doi:10.1038/s41467-021-25424-7)

Fig. 1f

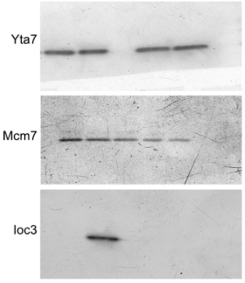

Fig. 3b

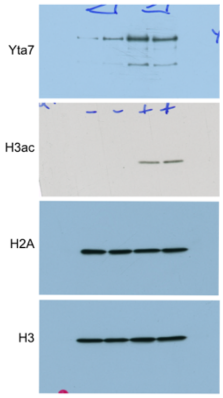

Fig. 3c

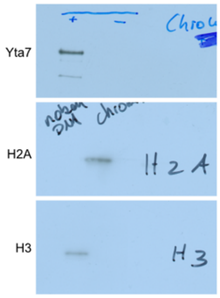

Fig. 3e

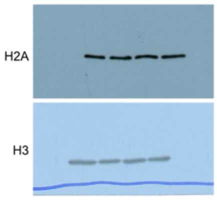

Fig. 5b

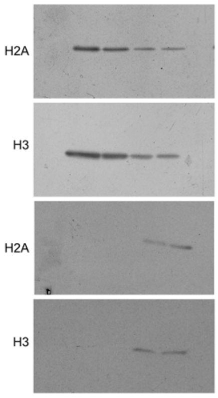

Fig. 5c

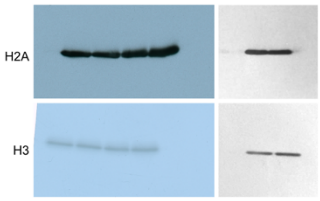

Supplementary Fig. 1b

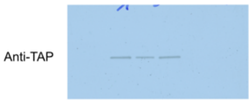

Supplementary Fig. 1c

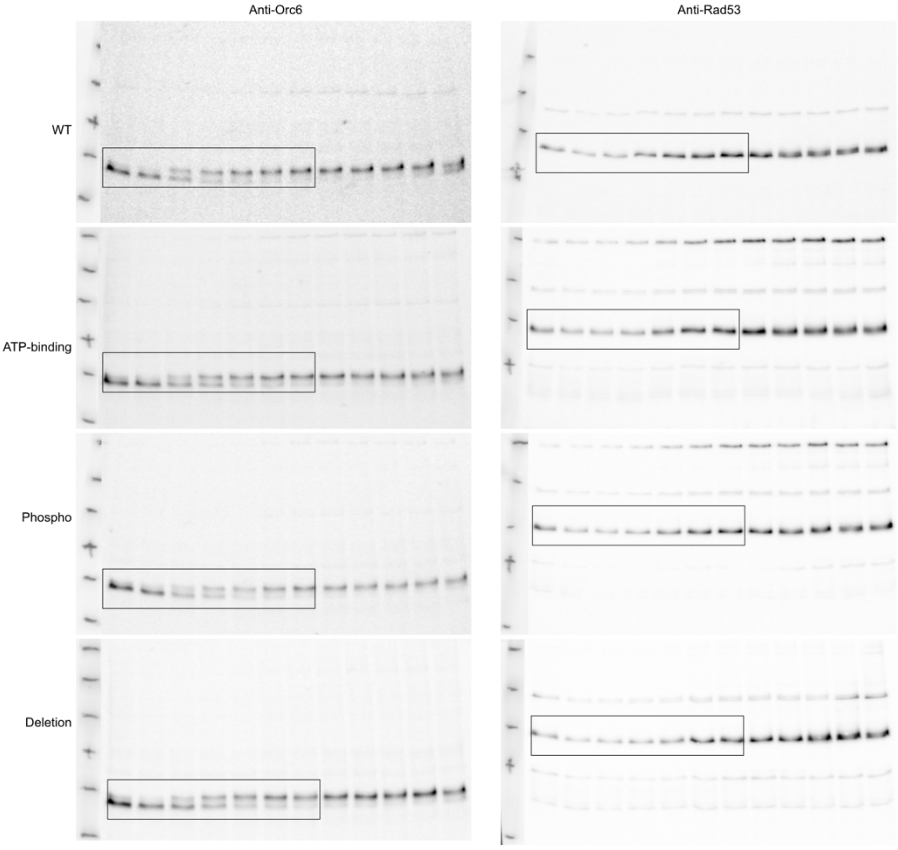

Supplementary Fig. 4b

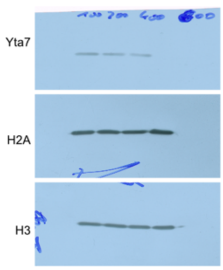

Supplementary Fig. 4c

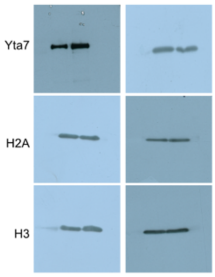

Supplementary Fig. 6

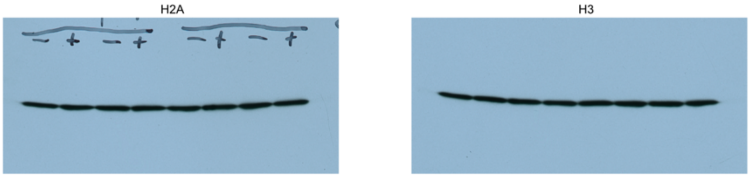

Supplementary Fig. 9

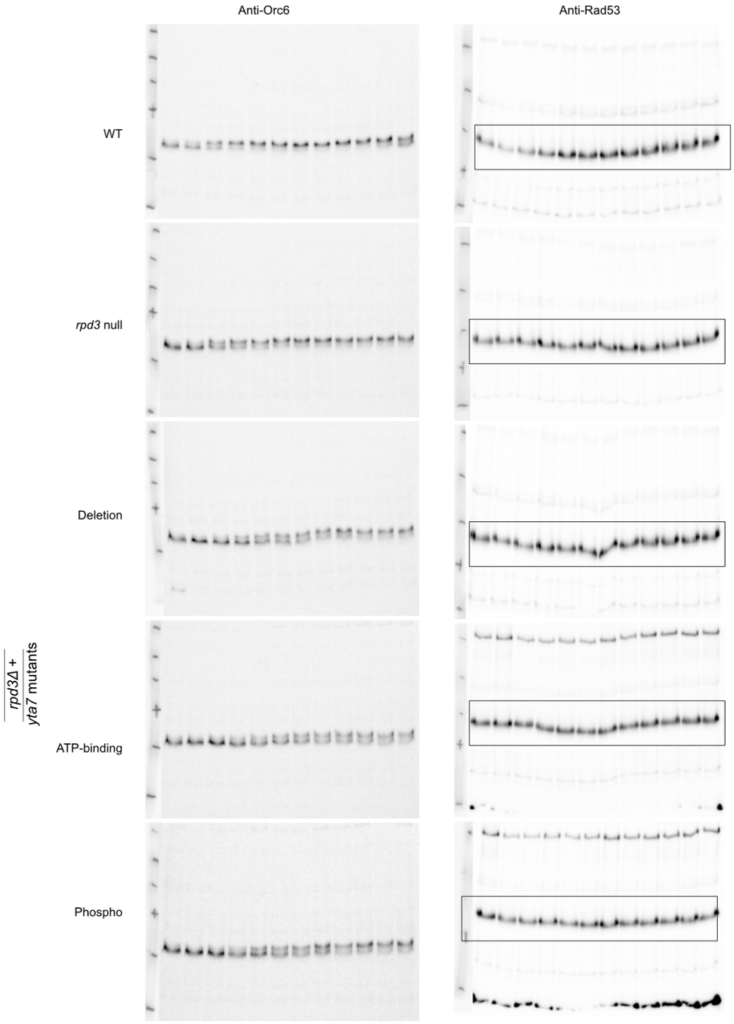

Supplement: Supplementary file 4 — Source Data [file 41467_2021_25424_MOESM4_ESM.zip › Kurat Source data/Uncropped blots.pdf]
